# Handgrip Strength and White Matter Integrity in Midlife Women at Risk for Alzheimer’s Disease

**DOI:** 10.1093/geroni/igaf122.3780

**Published:** 2025-12-31

**Authors:** Chadsley Wessinger, Jennifer Etnier, Samantha DuBois, Samuel Kibildis, Jarod Vance, Brittany Armstrong, Laurie Wideman, Alexis Slutsky-Ganesh

**Affiliations:** University of North Carolina at Greensboro, Greensboro, North Carolina, United States; University of North Carolina at Greensboro, Greensboro, North Carolina, United States; Appalachian State University, Boone, North Carolina, United States; Athens-Clarke County Unified Government, Athens, Georgia, United States; Longwood University, Farmville, Virginia, United States; University of North Carolina at Greensboro, Greensboro, North Carolina, United States; University of North Carolina at Greensboro, Greensboro, North Carolina, United States; North Carolina Agricultural and Technical State University, Greensboro, North Carolina, United States

## Abstract

Poor muscle health is an emerging risk factor for Alzheimer’s disease (AD), yet the pathways linking muscle health to AD risk remain unclear. Handgrip strength (HGS), a marker of muscle health, is associated with episodic memory (EM), the hallmark cognitive symptom of AD. White matter microstructural integrity (WMI) is also associated with EM and implicated in AD pathology. While HGS is associated with global WMI, it is unclear if it specifically relates to WMI in tracts critical for EM, like the fornix and hippocampal cingulum (CGH). Given that women are disproportionately affected by age-related muscle loss and AD, investigating these relationships in women may help uncover a mechanistic link between muscle health and AD risk in this population. Participants (N = 18 women, Mage=55.7±7.0, 66% post-menopause) completed HGS using a handgrip dynamometer; operationalized as the sum of the maximum of two trials per hand. Diffusion tensor imaging was used to estimate fornix and CGH fractional anisotropy (FA), mean diffusivity (MD), and axial diffusivity (AxD) as WMI measures. Linear regression models tested the extent to which HGS predicted WMI. The overall model predicting fornix AxD was significant (p=.026). Greater HGS predicted lower fornix AxD (p=.043), whereas no significant associations were observed between HGS and other WMI measures (p’s>.08). This suggests that greater muscle strength is predictive of more favorable WMI, (i.e., lower fornix AxD, reflecting less diffusion perpendicular to axonal fibers), suggesting better axonal integrity. Future work should use longitudinal designs to investigate if changes in muscle health predict changes in WMI.

